# Obesity: A Growing Multifaceted Problem

**DOI:** 10.5935/abc.20150133

**Published:** 2015-11

**Authors:** Paula S. Azevedo, Marcos F. Minicucci, Leonardo A. M. Zornoff

**Affiliations:** Departamento de Clínica Médica – Faculdade de Medicina de Botucatu – Universidade Estadual Paulista Júlio de Mesquita Filho – UNESP, Botucatu, SP – Brazil

**Keywords:** Obesity / complications, Overweight / physiopathology, Hypertension, Dyslipidemias, Public Health

Overweight and obesity are defined as body mass index (BMI) of 25-29.9 kg/m^2^ and ≥ 30 kg/m^2^, respectively. It is an alarming public health problem worldwide. Brazilian data show that 52.5% of Brazilians are overweight, and 17.9%, obese^1^. In the USA, 35% of the adults are obese^2^.

One major problem of obesity is its common association with arterial hypertension, dyslipidemia, insulin resistance and diabetes in the metabolic syndrome context^3,4^. The adipose tissue plays a relevant role in the secretion of inflammatory and humoral mediators, which can favor the increase in blood pressure and insulin resistance^5^. This area has witnessed several advances, mainly related to epigenetic changes. Obese individuals, for example, show DNA hypermethylation in a specific region that encodes adiponectin, favoring lower secretion of that compound and exacerbating metabolic disease^6^.

Obesity, either independently or not, is recognized as an important risk factor for cardiovascular disease and heart failure^7,8^. However, not all aspects of that association have been clarified. For example, a Brazilian national case series of 4050 asymptomatic patients undergoing exercise stress echocardiography has shown a greater prevalence of hypertension, diabetes, dyslipidemia, family history and sedentary lifestyle among obese individuals. However, on multivariate analysis adjusted for confounding factors, obesity has not associated with myocardial ischemia^9^. On the other hand, in patients with acute coronary syndrome, the abdominal circumference measure has associated with recurring angina, but not with other complications during hospitalization^10^.

Regarding the association of obesity and heart failure, in a case series with participants of the Framinghan study, an elevation of 1 kg/m^2^ has increased by 5% the risk of developing heart failure^7,11^. In fact, cardiac remodeling, clinically detected as left ventricular hypertrophy and left atrial enlargement, is observed in obese individuals^12,13^. There are several hypotheses relating obesity to cardiac remodeling, and consequently to myocardial dysfunction. Experimental studies have suggested the participation of insulin resistance, altering the energetic metabolism and favoring the intracardiac accumulation of lipids, a phenomenon described as lipotoxicity^12^. In addition, changes in calcium transport, in collagen and in epicardial fat have been reported as potential mechanisms responsible for obesity-induced cardiac remodeling, associated with myocardial dysfunction^12,14,15^. It is worth noting that genetic changes, such as ALK7 gene polymorphism, which encodes the TGF-beta receptor, can be related to ventricular remodeling in patients with metabolic syndrome^16^. On the other hand, experimental studies have not identified the participation of the glycolytic pathway^17^ or imbalance between phosphorylated and total phospholamban^18^, but have reported a reduction in the expression of type-I collagen in high-fat diet-induced obese rats^19^. Another important, although less studied, aspect is the possibility of right ventricular remodeling, caused by pulmonary hypertension, resulting from the restriction imposed by obesity or coexistence of obstructive sleep apnea syndrome^12,20^. Thus, the precise pathophysiological mechanisms associated with remodeling in obesity remain to be elucidated.

However, in some clinical situations, patients with overweight and mild obesity have had fewer hospitalizations and lower mortality as compared to those with lower BMI^7,21-23^. The same apply to the triceps skinfold, whose values greater than 20 mm have been associated with lower mortality^24^. This has been known as the paradox of obesity, and, although the mechanisms involved are unknown, the following hypotheses have been proposed: the U-shaped behavior, in which the extremes have higher risks; obese patients would be on more optimized treatments; and obese individuals could have a higher metabolic reserve to offset the catabolic changes of a chronic disease, such as heart failure^7^.

Another point that has been gaining attention recently is the overweight of children and adolescents. A Brazilian study, assessing 4609 children between 6 and 11 years of age, has shown that 24.5% of them are overweight^25^. In addition, overweight children and adolescents have a higher prevalence of arterial hypertension and dyslipidemia^13,25,26^. This context promotes the early increase of the cardiovascular risk, suggested by the correlation of BMI and insulin resistance, left ventricular mass index and intimal layer thickness of the common carotid artery^13^.

Finally yet importantly, the management of the obese patient poses another challenge. Weight regain induced by complex interactions between the hormonal and neuronal pathways, which regulate food intake, lifestyle, food pattern and genetic characteristics, hinders the continuous therapeutic approach of the obese patient^27^. Thus, medications or bariatric surgeries are accepted as useful to reduce BMI^27^. However, it is worth noting that the ultimate objective of the treatment is change in lifestyle and use of strategies to keep the patient motivated to loose weight^28,29^.

Regarding obesity and its association with cardiovascular disease, there are still many gaps to be filled. Likewise, obesity remains a priority for future studies, both clinical and experimental, in an attempt to better understand its pathophysiological mechanisms and its clinical repercussions.
